# Surgical-orthodontic approach for facial rejuvenation based on a reverse facelift

**DOI:** 10.1186/s40510-019-0287-8

**Published:** 2019-08-26

**Authors:** Carmen Lorente, Federico Hernández-Alfaro, Maria Perez-Vela, Pedro Lorente, Teresa Lorente

**Affiliations:** 1 Department of Human Anatomy and Histology, University of Zaragoza, Zaragoza, Spain; 2 Lorente Orthodontic Clinic, Paseo Constitución 29, 50001 Zaragoza, Spain; 3 Institute of Maxillofacial Surgery, Teknon Medical Center Barcelona, Barcelona, Spain; 4 Department of Oral and Maxillofacial Surgery, Universitat Internacional de Catalunya, C/Vilana no. 12, Despacho 185, 08022 Barcelona, Spain

**Keywords:** Facial rejuvenation, Soft and hard tissues, Orthognathic surgery, Facial esthetic, Reverse facelift, Maxillomandibular advancement (MMA)

## Abstract

**Background:**

Orthodontic treatment combined with a maxillomandibular advancement (MMA) can be an effective option for patients who need not only corrected occlusion but also facial rejuvenation. In this case series, two patients underwent orthodontic treatment and bimaxillary orthognathic surgery involving MMA, one of them with a counterclockwise rotation of the occlusal plane (OP).

**Findings:**

In both cases, the face was rejuvenated, a functional occlusion was established, and the posterior airway space (PAS) was widened.

**Conclusions:**

The facial mask ages three dimensionally. MMA should be offered to patients who have insufficient skeletal projection and are considering improving their facial appearance beyond just correcting a malocclusion problem. The reverse facelift provides more soft-tissue support, resulting in mid- and lower-face rejuvenation.

## Background

Facial aging is a dynamic lifelong process that is the consequence of many mechanisms involving the “facial mask” (skin, fat, ligaments, muscles, and periosteum) and the bone structure of the face [1].

Wrinkles appear on the skin, soft tissues migrate downward due to the effects of gravity and adipose and muscular tissue atrophy, and skeletal changes are generated by resorption. These first three processes affect the aging of the soft tissues, whereas the last affects the hard tissue [2–4].

During the first years of rejuvenation treatment, known as the *cutaneous period* (1900–1970), the techniques applied focused on the changes of the skin layer. The *Superficial Musculo-Aponeurotic System* (SMAS) *period* followed (1970–1980), in which Skoog introduced the concept of subfascial dissection [5]. According to this new technique, in addition to tightening and excising superfluous skin, the subcutaneous fat layer with its fascia, as well as the cutaneous muscles of the neck and face, are repositioned. After that, Mitz and Peyronie described the anatomical SMAS in the parotid and cheek area, which rapidly became the standard technique [6].

As the years went by, facelifting reached deeper layers, which led to the *deep plane period* (1980–1991). Tessier performed a subperiosteal dissection via a coronal incision of the malar bones, zygomatic arches, and orbital margins. Subperiosteal dissection was not only useful to lift the facial mask, but also for remodeling the orbital margins and obtain bone grafts, improving the results compared with previous techniques [4].

During these three periods, the facial aging process had only been understood as an increase in the number of wrinkles on the face and sagging with soft-tissue flaccidity. It was not until the present period when surgeons started addressing the volume loss of both the soft and hard tissues. Beyond the fat component, volume loss is also a consequence of continuous remodeling of the facial skeleton with time [7], with the most extensive changes at approximately 50 years of age in both men and women. Levine et al. [8] reported a longitudinal cephalometric analysis of midfacial growth in adults to determine the role of bone in facial aging leading to the *volumetric period* (1991–today), which is characterized by returning lost volume, mainly due to the loss of adipose tissue and bone resorption, to the face through a refined intervention with reduced scars. Fat grafting for facial filling has become popular [9–11], and a new philosophy based on facial skeletal expansion is now a matter of interest [1, 12]. It is now well known that a treatment based only on pulling, lifting, or repositioning soft tissue tends to produce a flattening effect of the structure being treated, often making it advisable to include anteroposterior (AP) dimension augmentation techniques [13].

Under-projection of the facial bones is usually associated with a lack of support at the facial mask level. Often, this condition is overlooked and incorrectly managed with conventional facelift soft-tissue techniques. It is paramount to establish an adequate diagnosis that allows the surgeon to decide which tissues (hard/soft) should be moved and in which direction to create an increase in volume. An important pillar in our field is orthognathic surgery, which is essential to achieve this effect known as “reverse facelift.” Maxillomandibular advancement (MMA) is the most effective procedure to achieve this goal [14]. The skeletal advancement moves forward the attached musculature, tissues, and tendons, improving the facial appearance and indeed increasing the pharyngeal airway space (PAS) [15]. In fact, MMA has been reported as the surgical treatment for patients with obstructive sleep apnea syndrome (OSAS) [15, 16].

Nevertheless, in contemporary “orthofacial” surgery, a holistic approach must be embraced, combining hard and soft tissue management to achieve the ideal facial harmonization at all tissue levels [17].

In the authors’ protocol, a vertical plane through the soft-tissue nasion serves to evaluate the AP position of the skeletal lower third of the face and, hence, to diagnose a possible deficiency at the bone level. The plane should be traced perpendicular to the floor, with the patient in the natural head position. To be considered adequate, all the soft-tissue profile of the patient in the lower third should be at or in front of this plane. Moreover, the upper incisor (provided it has the appropriate torque) should also be at or in front of this reference plane [18].

We present two ortho-surgical cases that underwent MMA to improve not only a malocclusion problem, but also the facial appearance, as it was the main complaint in both situations. The aim of this case series was to show that orthodontic treatment combined with a MMA can be an effective option for patients who require not only an occlusion correction but also a rejuvenation effect. The orthodontist should be aware that in patients presenting facial aging, all the different treatment options should be offered, informing patients of the effects of facial rejuvenation that MMA surgery can provide.

## Materials and methods

### Patient 1

#### Diagnosis and etiology

The patient, a 51-year-old woman with no significant medical history, visited the clinic in Zaragoza, Spain, with the main complaint of improving her occlusion, but also willing to enhance her facial esthetics.

The facial photographs showed a Class II skeletal pattern due to a severe mandibular hypoplasia, lack of support of facial tissues, and sagging of the neck tissue with a short throat length. The occlusal plane (OP) was slightly canted, and the mandibular sulcus contour was accentuated, something that is usually secondary to a Class II deep bite.

The intraoral examination showed that the overjet and the overbite were 7.2 mm and 5.8 mm, respectively, with an accentuated curve of Spee and proinclination of the lower incisors. The canine and molar keys showed asymmetric Class II relationships on the left and right side with a shifted inferior midline.

**Fig. 1:**
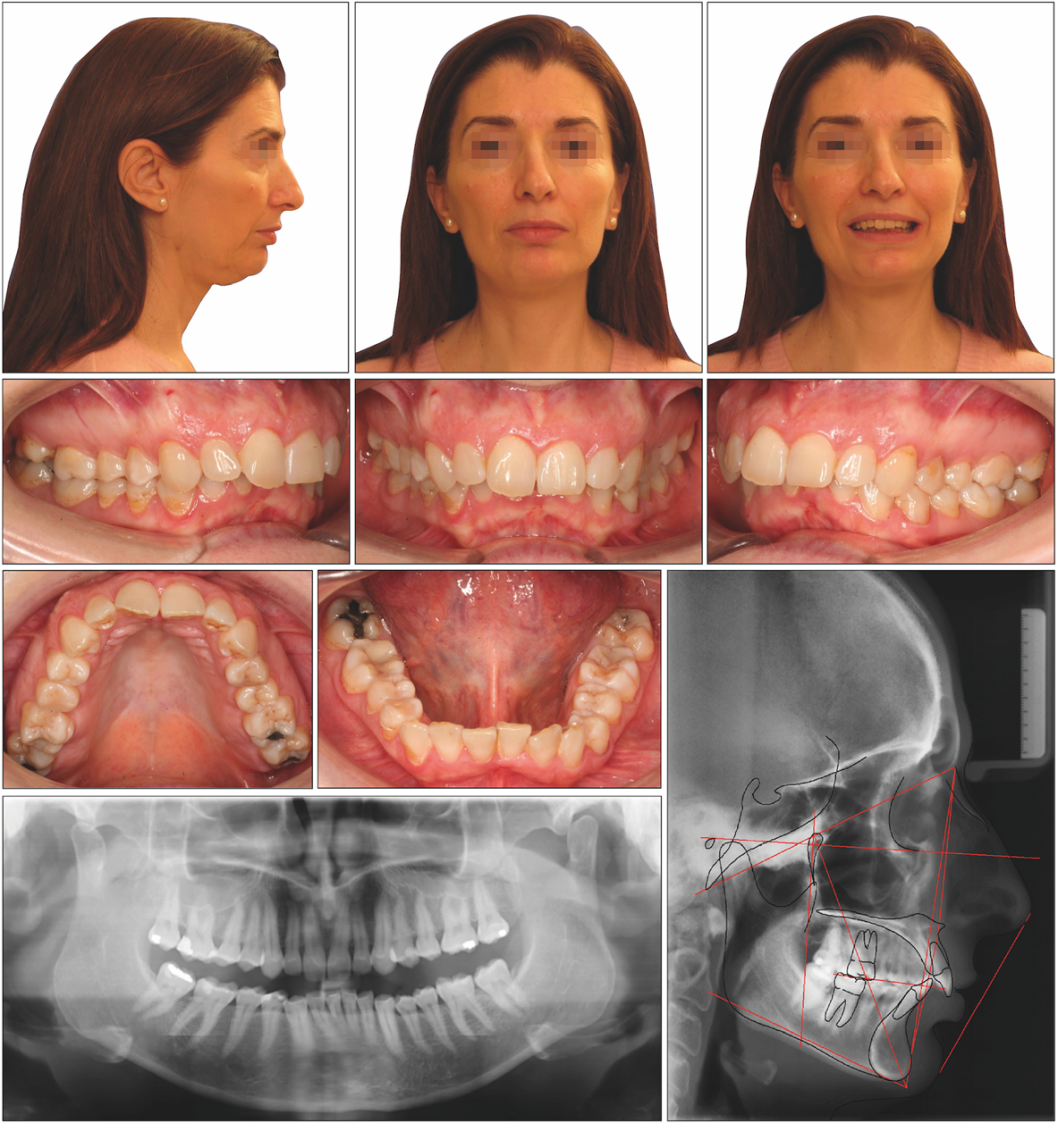
Pretreatment records of patient 1

The cephalometric analysis showed that the patient had a hypodivergent facial pattern (lower facial height 60.9; facial axis 83.7) with a severe skeletal Class II relationship and an increased ANB angle (4.9°). The mandibular incisors were moderately inclined buccally (IMPA 107.8°) to compensate for the skeletal sagittal discrepancy (Table 1). The initial posterior airway space (PAS) was 1.25 mm. The panoramic radiograph showed all teeth, except for the third molars (Fig. 1).

**Table 1:** Cephalometric measurements of patient 1

Measurement	Norm	Pretreatment	Posttreatment
SNA (°)	82	75.9	78.3
SNB (°)	80	70.9	76.5
ANB (°)	2	4.9	1.8
Interincisal angle (°)	130	119.8	107.7
Mx1 to A-Po (°)	28	29.2	25.2
Md1 to A-Po (°)	22	31	27.4
Facial axis (NaBa-PtGn) (°)	90	83.7	87.7
IMPA (°)	95	107.8	94.8
Lower facial height (ANS-Me) (mm)	66	60.9	61
Midface length (Co-A) (mm)	93.2	83.0	85.3
Mandibular length (Co-Gn) (mm)	122.3	108.1	114.2

#### Treatment objectives

The following treatment objectives were established: (1) achieve appropriate anterior overbite and overjet relationships, (2) obtain Class I canine and molar relationships for the recovery of occlusal function, (3) level the curve of Spee, (4) correct the dental midline deviation, and (5) improve the facial profile with a better facial appearance.

Because the maxillomandibular discrepancy was severe, the surgical approach was the only option to achieve the previous objectives.

#### Treatment alternatives

Before offering the patient the treatment options, the clinicians assessed the best way to level the lower curve of Spee without increasing the mandibular incisor inclination. One of the possibilities was the extraction of the first two lower premolars, which would allow distalizing the mandibular arch, creating more overjet. The other option was distalization of the third and fourth quadrants with miniscrews. Since the patient did not have lower third molars, the second option was chosen, so that the upper second molars were not left without support.

After deciding the best orthodontic mechanics to flatten the curve of Spee, the following treatment alternatives were presented to the patient. As a first option, a mono-maxillary orthognathic surgery was proposed to the patient. Once a greater overjet was achieved with skeletal anchorage, a mandible advancement could be performed. Although this option would have been enough to correct the occlusal problem, the bimaxillary skeletal retrusion would have remained after surgery without achieving major changes in the facial profile. Due to this reason, bimaxillary orthognathic surgery was the second alternative. A MMA was proposed to increase the facial volumization. As both alternatives were orthodontic-surgical treatments, the patient chose the second option, since it provided greater improvement of facial esthetics.

#### Treatment progress

The treatment plan included fixed orthodontic appliances with 0.022 in. × 0.028 in. slot metal brackets (Roth prescription; Straight-Wire Synthesis; Ormco, Glendora, Calif). Two miniscrews of 10.0 mm in length and 2.0 mm in diameter (VectorTAS; Ormco, Glendora, Calif) were inserted in the mandibular buccal shelf to allow the distalization of the mandibular arch and create enough overjet for the mandibular advancement. The activation of both miniscrews was performed every 2 weeks with elastic thread connected from the miniscrew’s head to the brackets.

After 12 months of treatment, passive rectangular (0.021 × 0.025-in.) stainless steel wires with surgical hooks were inserted in both arches before orthognathic surgery.

**Fig. 2:**
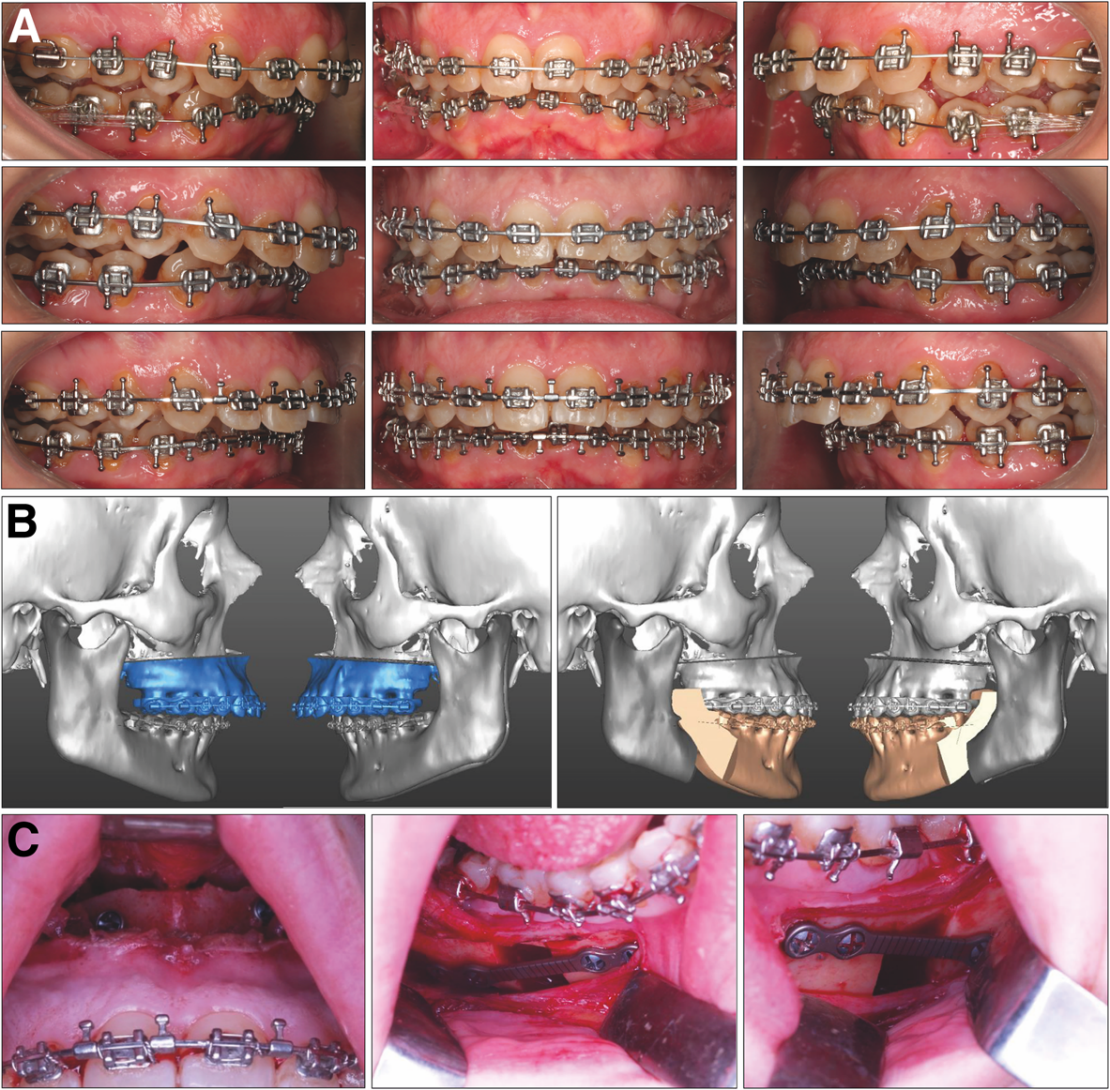
**a** Presurgical orthodontic decompensation with mandibular miniscrews. **b** Surgical treatment plan generated by SimPlant software. **c** Trans-surgical photographs showing septoplasty, LeFort I osteotomy, and bilateral sagittal split osteotomy for mandibular centering

The MMA was planned with the SimPlant Pro Crystal software (Materialise, Leuven, Belgium). Surgery was performed including bilateral sagittal split osteotomy procedures using the intermediate splint to achieve mandibular centering. A segmented LeFort I maxillary osteotomy with 4.45 mm of advancement was performed concomitantly with a septoplasty, and an advancement of 13.07 mm was performed in the mandible (Fig. 2).

In the postsurgical orthodontic treatment, brackets were rebonded, and the patient was instructed to use Class II elastics (Masel®, 1/8-in, 6.0-oz) to establish correct intercuspidation.

#### Treatment results

After 18 months of active treatment, the patient was debonded. Retention included an Essix retainer in the upper arch and a fixed canine-to-canine lingual retainer in the lower arch.

The posttreatment photographs of the patient and her smile confirmed good esthetics and dental relationships. The face results showed a change in shape and fullness, with a higher definition of her chin and less pronounced nasolabial falls.

The posttreatment intraoral photographs show a Class I molar and canine relationship on the left side and a slight Class II tendency on the right side with good interdigitation of the lateral segments, competent lips, and ideal overjet and overbite.

The panoramic radiograph confirmed the correct parallel root positioning. The lateral cephalometric radiograph and tracing confirmed the dental and skeletal changes after treatment. The most significant cephalometric changes were the increase of the SNA of 7.9° and the SNB of 10.4°, with a counterclockwise rotation of the maxillomandibular complex of 8°, probably due to the intrusion effect of the miniscrews. In fact, the curve of Spee was able to level, improving the inclination of the lower incisors (Md1 to A-Po 27.4°) due to the distalization achieved with the miniscrews (Table 1).

**Fig. 3:**
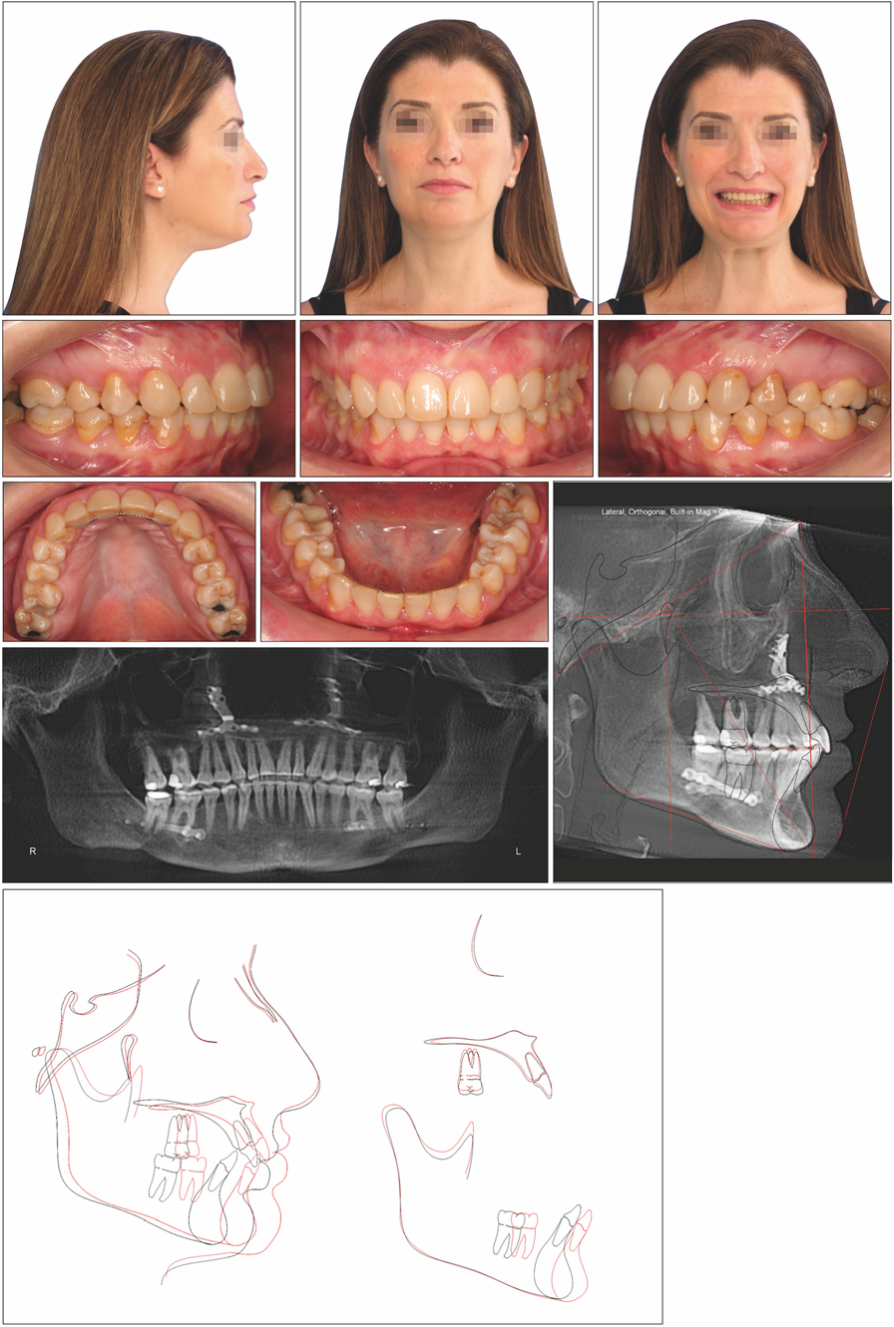
Posttreatment records and superimposition of tracings before treatment (*black line*) and after treatment (*red line*) of patient 1

The superimposition showed improvement in the positioning of the maxillary and mandibular incisors (Fig. 3).

Not only both jaws improved their positions, but also the preoperative PAS of 1.25 mm increased, obtaining a final value of 16.75 mm. It was measured by CBCT as described by Hernández-Alfaro et al. [19]. After the MMA, the total volume of the pharyngeal airway space increased from 31.515 to 39.814 mm^3^.

### Patient 2

#### Diagnosis and etiology

A 37-year-old woman who wanted an improvement of her facial appearance came to our clinic. She reported that she had undergone two previous 2-year orthodontic treatments, the first one with four second premolar extractions. After these two treatments, the patient continued to dislike her profile result. The facial photographs showed bimaxillary retrusion with perioral wrinkles due to a loss of bone support accentuated by the previous premolar extractions. The upper lip was retruded − 6.6 mm and the lower lip − 11.0 mm in relation to a perpendicular line from subnasale (Sn). The intraoral photographs and dental casts showed Class I molar and canine relationships.

**Fig. 4:**
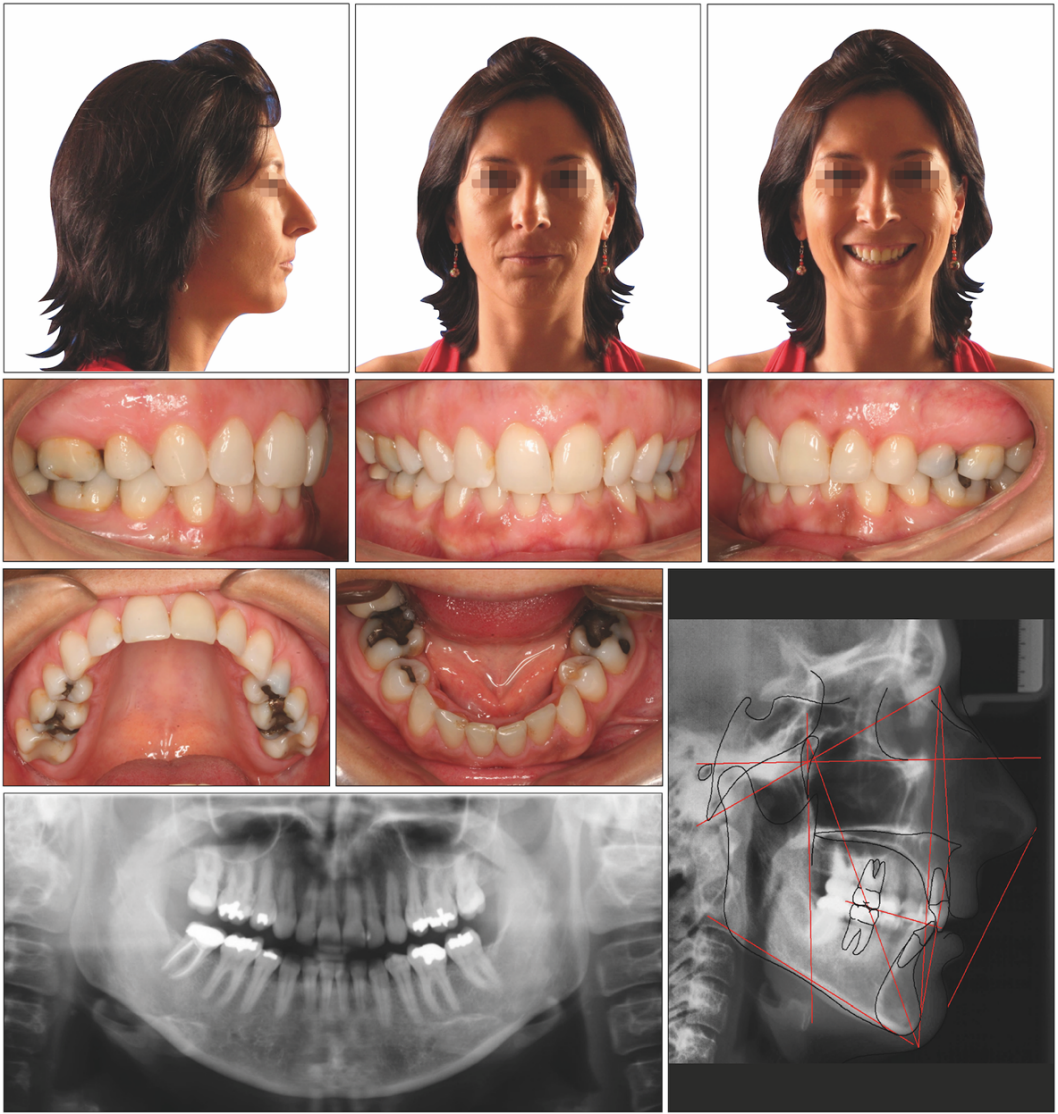
Pretreatment records of patient 2

The cephalometric analysis showed a skeletal Class II pattern (ANB; 8.5°) with a clockwise rotation of the OP to sella-nasion of 23.2° (Fig. 4).

#### Treatment objectives

Treatment objectives were as follows: (1) correct subtle dental malpositions preserving a functional occlusion, (2) improve the facial profile, and (3) rejuvenate the face by increasing the volume loss in the middle and lower third.

#### Treatment alternatives

As the patient’s main objective was achieving esthetic facial changes, a nonsurgical orthodontic approach was discarded. Therefore, the option offered was an orthodontic treatment combined with bimaxillary orthognathic surgery involving MMA with a counterclockwise rotation of the OP.

#### Treatment progress

Full fixed preadjusted appliances with 0.022 × 0.028-inch slots were placed in both arches. After 6 months of treatment, passive rectangular 0.021 × 0.025-inch stainless steel wires with surgical hooks were inserted in both arches before orthognathic surgery.

**Fig. 5:**
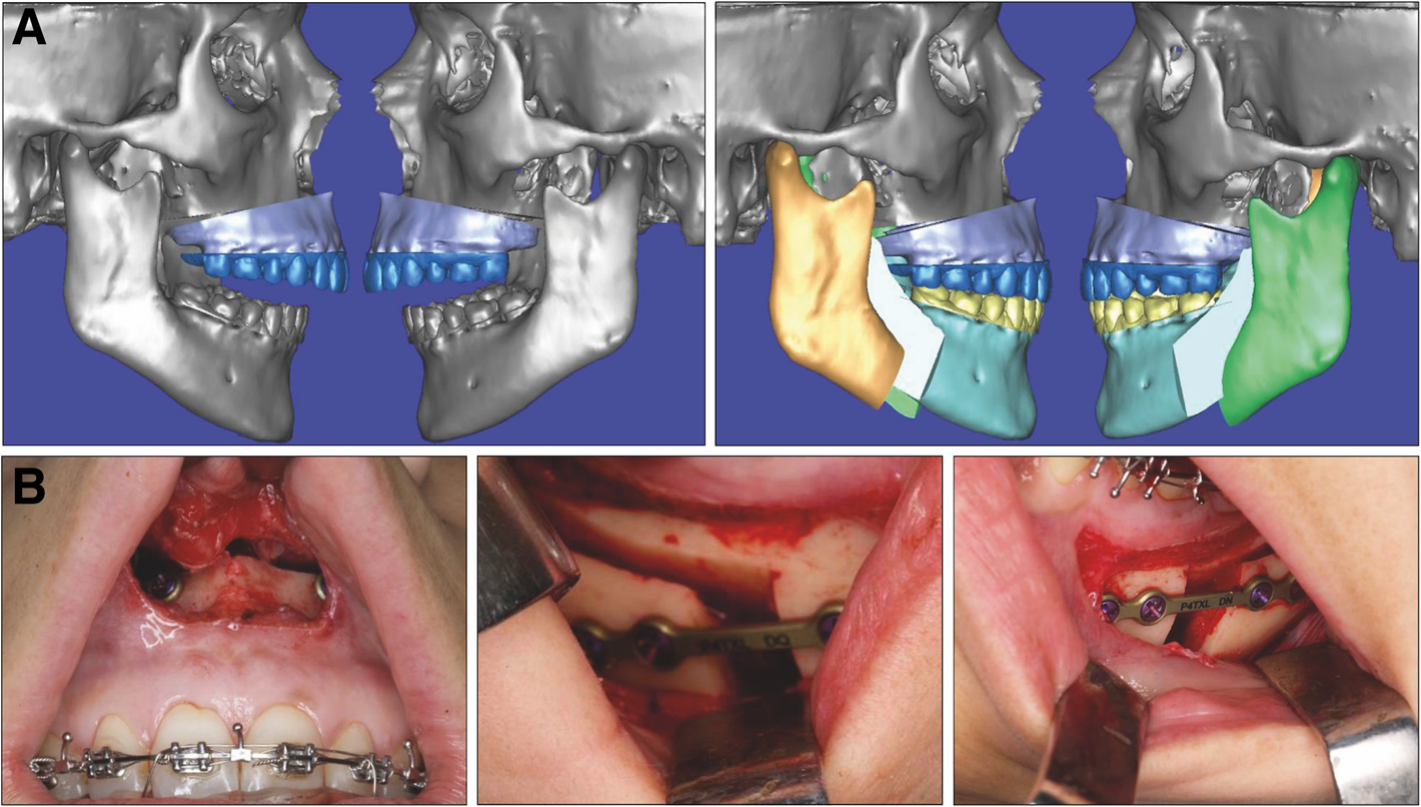
**a** Surgical treatment plan generated by SimPlant software. **b** Trans-surgical photographs showing LeFort I osteotomy and bilateral sagittal split osteotomy for mandibular centering

The MMA was planned with the SimPlant software. Surgery was performed including bilateral sagittal split osteotomy procedures using the intermediate splint to achieve mandibular centering. A segmented LeFort I maxillary osteotomy with 7.78 mm of advancement was performed. The mandible was also counterclockwise rotated 10.47° and an 18.07-mm advancement was performed (Fig. 5).

In the postsurgical orthodontic treatment, the patient was instructed to use Class I elastics to establish correct intercuspidation.

#### Treatment results

After less than 1 year of active treatment, the patient was debonded. Retention included Essix retainer in the upper arch and fixed canine-to-canine lingual retainer in the lower arch.

The posttreatment records showed that the treatment objectives were achieved. The face showed an increase in facial volume leading to a reduction of the perioral wrinkles and a pleasing facial esthetic.

Posttreatment intraoral photographs showed bilateral Class I molar and canine relationships with good overjet and overbite.

The radiographs and tracing confirmed correct dental and skeletal changes after treatment. There was an improvement of 7.5° in the inclination of the maxillary incisors and a reduction of 4.8° of the mandibular incisors (Table 2). After the surgery, the upper lip was − 1.4 mm and the lower lip − 3.5 mm in relation to a perpendicular line from Sn.

**Table 2:** Cephalometric measurements patient 2

Measurement	Norm	Pretreatment	Posttreatment
SNA (°)	82	85.8	91.3
SNB (°)	80	77.4	83.1
ANB (°)	2	8.5	8.2
Interincisal angle (°)	130	141.0	138.3
Mx1 to A-Po (°)	28	11.1	18.6
Md1 to A-Po (°)	22	27.9	23.1
Facial axis (NaBa-PtGn) (°)	90	79.9	85.8
IMPA (°)	95	96.2	88.4
Lower facial height (ANS-Me) (mm)	66	69.9	67.5
Midface length (Co-A) (mm)	93.2	81.1	85.7
Mandibular length (Co-Gn) (mm)	122.3	111.7	117.5

**Fig. 6:**
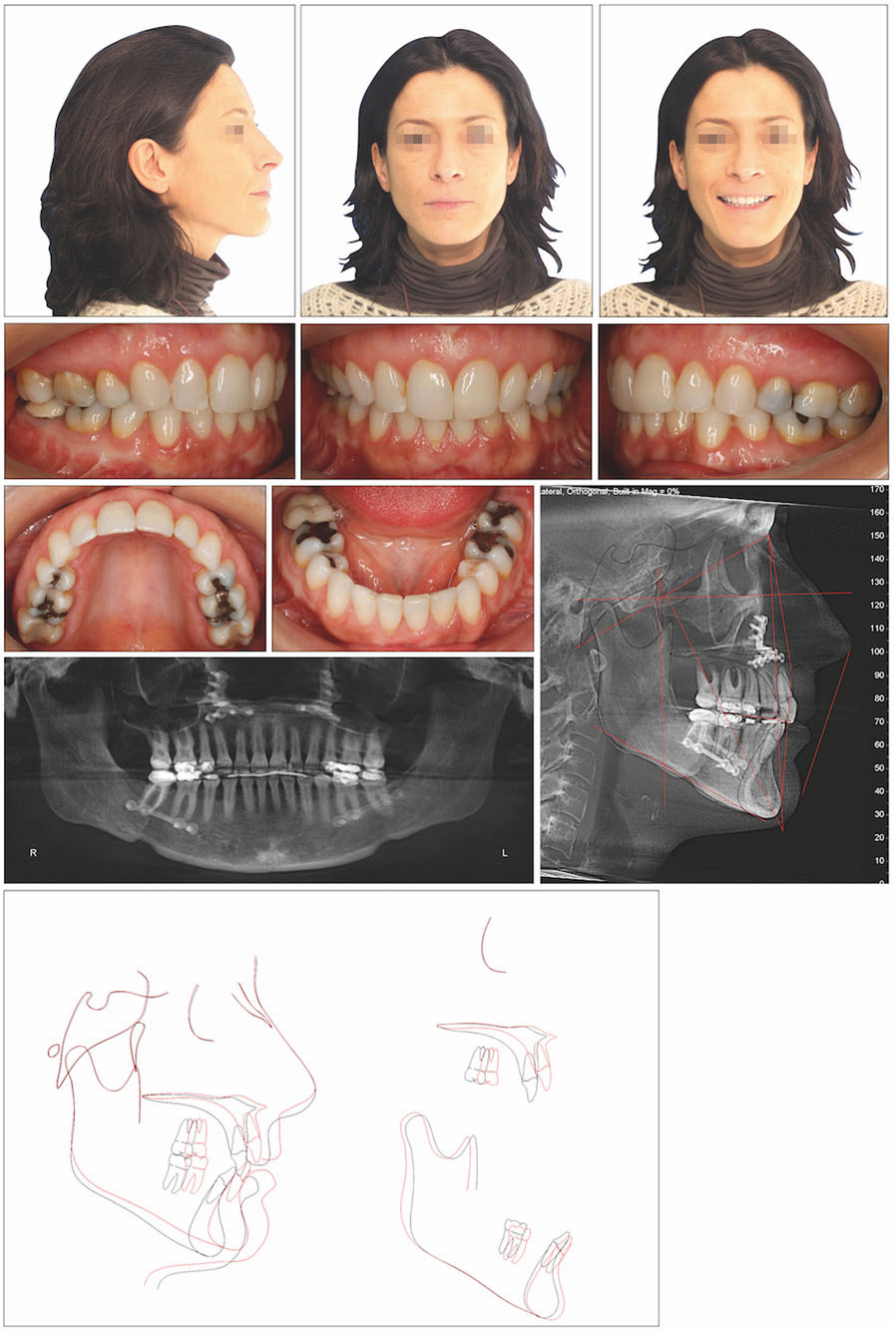
Posttreatment records and superimposition of tracings before treatment (*black line*) and after treatment (*red line*) of patient 2

The superimposition showed an important and precise rotation of the maxillomandibular complex to enhanced facial esthetics, resulting in an OP of 14.8° (Fig. 6).

In this case, the PAS also improved notably. After the MMA, the total volume of the pharyngeal airway space increased from 23.767 to 55.665 mm^3^.

The postoperative PAS was 7.25 mm, having achieved an increase of 2.5 mm.

## Discussion

The demand for esthetic treatments is constantly growing [20]. This increased demand also affects the orthodontic field, with more patients who do not only seek optimal functional results, but also a global goal that encompasses the improvement of physical appearance [21].

Over the years, rejuvenation therapy has changed the priorities of the facial components, giving more importance to the volume at the level of the middle third [22]. An improvement in facial shape remains the primary goal in facial rejuvenation [23].

**Fig. 7:**
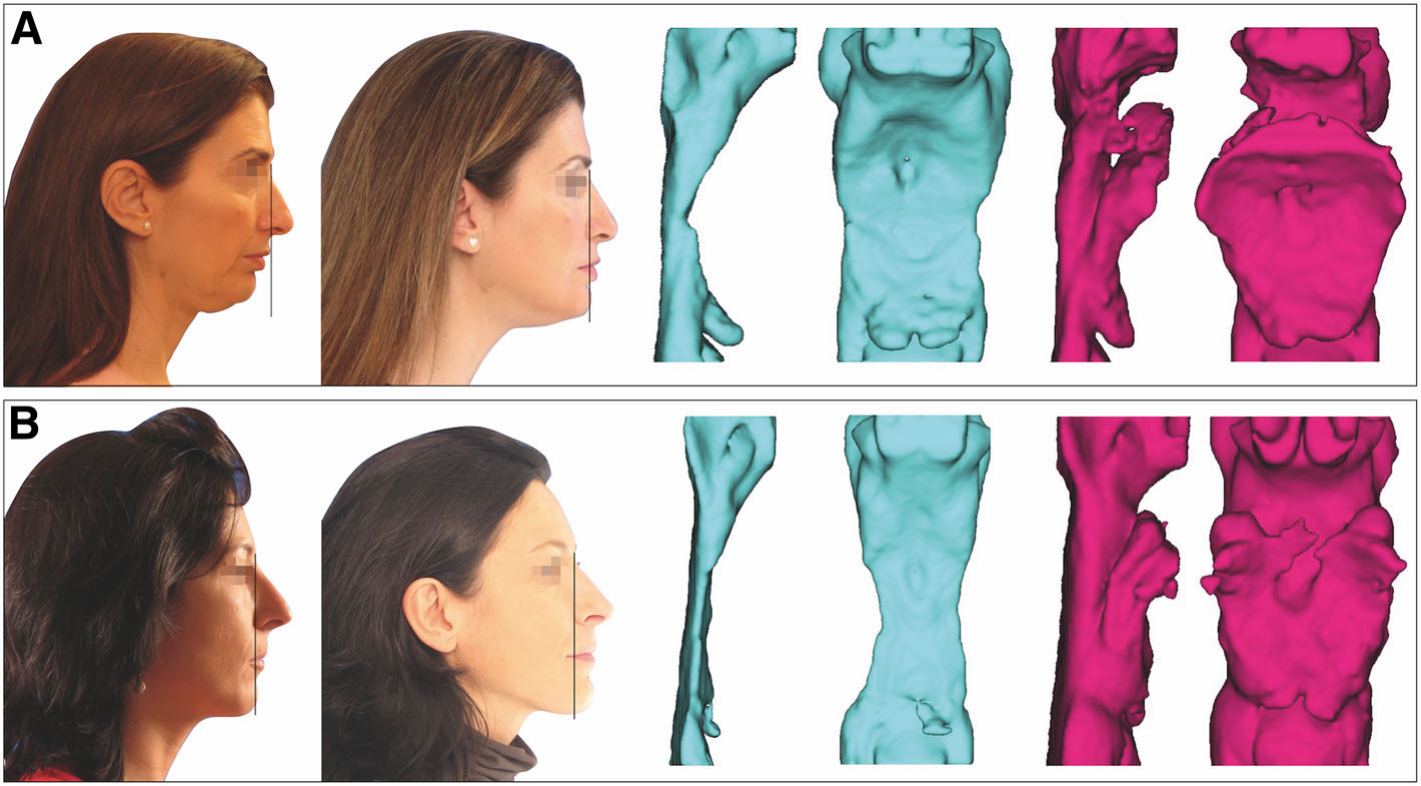
Pretreatment and posttreatment profile changes in relation to a vertical plane through the soft-tissue nasion and preoperative and postoperative posterior airway space and volume for patient 1 (**a**) and patient 2 (**b**)

In both patients, the degree of facial rejuvenation was achieved only by projecting the hard tissues (Fig. 7). Orthognathic surgery alone achieved the remarkable effect erasing the lines present in the perioral area of the second patient. On the other hand, often projection of hard tissues is not enough to reach the overall rejuvenation goal, and it should be associated with soft-tissues techniques. Indeed, in the first case, the patient did not accept the option of performing a facelift at the same time as the MMA, which is why a slight sagging of remaining tissue can be observed at the neck.

It is essential for the orthodontist to remember the importance of the role played by the loss of bone volume in facial aging when considering a treatment plan. This is especially true in the second case, where the loss of bone support was accentuated when the patient was previously orthodontically treated with premolar extractions.

In addition to the anti-aging effect, an increase in the PAS is usually observed after treatment (Fig. 7). In fact, MMA has shown to be the most effective surgical option for patients with airway obstruction during sleep [16]. A retroposition of the maxilla, the mandible, or both, can lead to a diminished airway volume that results in collapse. This concern has been widely demonstrated as a higher incidence of OSAS was recorded in patients who underwent a setback for the correction of mandibular prognathism [14–16, 19, 24]. On the contrary, when a MMA is performed, the soft palate, tongue, related musculature, and hyoid bone move forward, increasing the PAS, as observed in Arcuri et al.’s [14] study, where 16 patients with OSAS underwent MMA, finding a 81% of success with a mean difference between pre- and postoperative PAS of 7 mm.

In the two cases presented, different approaches could have been made if the only goal was to achieve a correct occlusion. The first case could have been solved with a mono-maxillary surgery, and the second one could have been achieved even without surgery. However, as the patients’ complaint was not only a correction of a malocclusion problem, but also an improvement of their facial appearance, bimaxillary surgery was required to achieve an increased projection of their face, providing a facelift effect.

It is crucial to identify the patient’s complaints and goals in their first visit to the clinic. When there is a loss of facial volume, it will have to be analyzed if it is due to adipose tissue atrophy or bimaxillary retrusion. In this last situation, an advancement of both jaws with orthognathic surgery will provide the necessary support for facial tissues, achieving facial rejuvenation.

## Conclusions

The facial mask ages three-dimensionally. If there is a lack of skeletal support, this process is further pronounced. Maxillomandibular advancement should be offered to patients who are diagnosed with bimaxillary skeletal retrusion or posterior divergence and who are considering improving not only their occlusion but also their facial appearance. The reverse facelift provides more soft-tissue support, resulting in mid- and lower-face rejuvenation.

The two cases presented benefitted from a good orthodontic result with an improved facial esthetic.

## Data Availability

Please contact the author for data requests.

## Abbreviations

A: A point, deepest bony point on the contour of the premaxilla below ANS
ANB: Angle between point A, B and point N
AP: Anteroposterior
B: B point, deepest bony point on the contour of the mandible above pogonion
IMPA: Angle between long axis of lower incisor and mandibular plane angle
MMA: Maxillomandibular advancement
OP: Occlusal plane
PAS: Posterior airway space
SMAS: Superficial musculo-aponeurotic system
SNA: Angle between sella, nasion, and A point
SNB: Angle between sella, nasion, and B point
